# What do infectious disease specialists think about managing long COVID?

**DOI:** 10.1017/ash.2023.519

**Published:** 2023-12-14

**Authors:** Maureen D. Lyons, Susan E. Beekmann, Philip M. Polgreen, Jonas Marschall

**Affiliations:** 1 Division of General Internal Medicine and Geriatrics, School of Medicine, OHSU, Portland, OR, USA; 2 Department of Medicine, Washington University School of Medicine, St Louis, MO, USA; 3 Departments of Internal Medicine and Epidemiology, University of Iowa, Iowa City, IA, USA; 4 Division of Infectious Diseases, Washington University School of Medicine, St Louis, MO, USA

## Abstract

This survey of infectious disease providers on long COVID care revealed a lack of familiarity with existing resources, a sentiment of missing guidelines, and scarcity of dedicated care centers. The low response rate suggests that infectious disease specialists do not consider themselves as the primary providers of long COVID care.

Long COVID (or post-acute sequelae of COVID-19, PASC) complicates an estimated 10–30% of non-hospitalized cases and 50–70% of hospitalized cases of acute SARS-CoV-2 infection.^(1)^ The work-up often requires multiple disciplines to collaborate, both for diagnostic evaluation and for offering symptomatic treatment.^(2)^ For this purpose, an infrastructure of so-called long COVID clinics has been developing,^(3)^ oftentimes under the supervision of a single medical specialty. Given that long COVID is a complication of an infection, the medical subspecialty of infectious diseases (ID) has frequently been involved in devising and running such clinics. Here, we intended to survey ID providers in North America regarding their role in managing long COVID and their perspective on the availability of resources. For this purpose, we used the Emerging Infections Network (EIN), which is a provider-based sentinel network funded by the Infectious Diseases Society of America and the Centers for Disease Control and Prevention (http://ein.idsociety.org/). Our implicit hypothesis was that ID providers are insufficiently equipped to provide long COVID care because neither infection work-up nor antiviral treatment play a role in current management.

We designed a questionnaire, tested it among peers, and sent it out to all 2,978 EIN listserv subscribers on 3 occasions between February 7 and February 26, 2023. The survey contained 8 questions, 7 of which were structured questions. There was an option to make additional open-text field comments.

The response rate was very low, with 117 of 2,978 providers who filled out the survey (3.9%). Of these 117, 46 stated that they did not care for long COVID patients, and we analyzed the responses of the remaining 71 providers (2.4% of 2,978). Most of these would see long COVID patients once a month or less often (50/71). Thirteen indicated they were specifically seeing long COVID patients. Only 15 out of 71 (21%) felt “very comfortable” in making a diagnosis of long COVID, and most thought the resources available to clinicians involved in long COVID care were inadequate (55%) or were unaware of such resources (18%). The management consists mostly of a case-by-case-based approach without standardization (55%), followed by behavioral education for energy conservation (44%). Access to a long COVID clinic was considered easy by only 17%, while 48% stated there was no dedicated clinic located nearby, and 35% highlighted that locally available clinics were not easily accessible.

The open-text field answers were notable for showing that long COVID is a “complicated syndrome, currently without a specialty home” and that it is not easy to provide “holistic care to patients in our existing system.” Also, the absence of straightforward definitions, national guidelines, and dedicated research was pointed out. Lastly, one provider argued that “this is a primary care issue with collaboration with any needed specialists.”

The low response rate, the small percentage of ID providers heavily involved in long COVID care, and some free text statements can be seen as key findings of our survey. Our interpretation is that long COVID—although originating from an acute infection—is not perceived to be in the wheelhouse of the infectious diseases subspecialty. Infectious disease providers may not consider themselves as well-equipped as a generalist such as primary care providers.^(4)^ Long COVID patients, however, are likely to benefit from designated points of generalist care, with access to pertinent medical specialties and rehabilitative services (physical therapy, occupational health, and mental health), in order to receive comprehensive management for their signs and symptoms. In addition, widespread healthcare worker burnout in the wake of the pandemic may contribute to a subjective saturation with COVID-19-related topics and have reduced survey participation.

A minority of responders considered themselves very comfortable in diagnosing long COVID, the resources for appropriate clinical care were mostly felt to be inadequate, and management seems to happen on a case-by-case base (i.e., non-standardized). This points to the general unease about what constitutes best practice in long COVID care. We speculate that similar findings would be encountered in other medical specialties. Also, there is a considerable need for more research in the field, as evidenced by a recent award by the Agency for Healthcare Research and Quality to study new models of delivery of long COVID care (https://www.ahrq.gov/coronavirus/long-covid-grant-awards.html).

In conclusion, our survey of infectious disease providers and their perspective on long COVID care suggested a lack of familiarity with existing resources, a sentiment of missing guidelines, and scarcity of dedicated care centers. In addition, the low response rate to this survey can be interpreted as ID providers not regarding their specialty as the primary point of contact for delivering long COVID care.
